# Vertrauen als Grundlage einer erfolgreichen institutionellen Risikokommunikation

**DOI:** 10.1007/s00103-022-03519-w

**Published:** 2022-04-05

**Authors:** Ortwin Renn

**Affiliations:** Institute for Advanced Sustainability Studies (IASS), Berliner Str. 130, 14467 Potsdam, Deutschland

**Keywords:** Vertrauenswürdigkeit, Glaubwürdigkeit, Fake News, Bedingungen für erfolgreiche Kommunikation, Verständigungsorientierte Dialogverfahren, Trustworthiness, Credibility, Fake News, Condoitions for successful communication, Deliberative methods of reaching mutual agreements

## Abstract

Der Erfolg der Krisen- und Risikokommunikation einer Institution beruht auf einer offenen und dialogorientierten Kommunikationspolitik sowie auf einer Kongruenz zwischen den Erwartungen, die ihr gegenüber gehegt werden, und deren Erfüllung. Zentral für die Verständigung mit anderen Akteuren und mit der Bevölkerung ist ein gegenseitiges Vertrauensverhältnis. In diesem Beitrag werden Faktoren beschrieben, die maßgeblich dafür verantwortlich sind, ob und in welchem Ausmaß Institutionen eine Vertrauensbasis und Glaubwürdigkeit schaffen können. Es wird diskutiert, wie eine vertrauensvolle Risikokommunikation auch in Krisenzeiten gelingen kann.

Gelingende Risikokommunikation ist an ein Verfahren gebunden, das Glaubwürdigkeit und Kompetenz durch Offenheit der Ergebnisse, überzeugende Vermittlung von wissenschaftlicher Evidenz, den Einbezug pluraler Werte und Kriterien sowie die Synthese von System- und Orientierungswissen vermittelt. Angesichts der Unsicherheit von Wissen und der Unübersichtlichkeit der Kommunikationsvorgänge in der Gesellschaft ist diese Aufgabe nicht leicht zu erfüllen. Trotz dieser Schwierigkeiten können Institutionen der Risikobewertung und -regulierung durch geschickte Koalitionsbildung mit Organisationen und Gruppen mit hohem Vertrauenspotenzial, durch transparente, ergebnisoffene Formen der Kommunikation und durch die Einbindung von Stakeholdern und betroffenen Individuen in das Risikomanagement Vertrauen aufbauen und über die Zeit verstetigen.

## Abstract (en)

The success of institutional crisis and risk communication is based on an open and dialogue-oriented communication policy as well as on a congruence between the expectations of all parties involved and their fulfillment. Central to communication with other actors and with the population is a mutual relationship of trust. This paper describes factors that are instrumental in determining whether and to what extent institutions can establish a basis of trust and credibility. It discusses how trustworthy risk communication can succeed even in times of crisis.

Successful risk communication is tied to a process that conveys credibility and competence through openness of results, convincing communication of scientific evidence, the inclusion of plural values and criteria, and the synthesis of system and orientation knowledge. Given the uncertainty of knowledge and the confusion of communication processes in society, this task is not easy to accomplish. Despite these difficulties, institutions of risk assessment and regulation can build trust and perpetuate it over time through skillful coalition-building with organizations and groups with high trust potential, transparent, open-ended forms of communication, and the involvement of stakeholders and affected individuals in risk management.

## Einleitung

In Zeiten von Bedrohungen der kollektiven Sicherheit oder Krisen erwarten die Menschen von Politik und gesellschaftlichen Entscheidungsgremien wirksame Maßnahmen zur Eindämmung der Risiken sowie eine vertrauenswürdige Kommunikation über die Hintergründe und Beweggründe für diese Maßnahmen [1]. Wie sehr diese Erwartung erfüllt bzw. enttäuscht werden kann, hat die Coronakrise allen deutlich vor Augen geführt [2]. Konnte man in der ersten Welle im Jahr 2020 noch von einem Schulterschluss zwischen Regierung, Wirtschaft, Zivilgesellschaft und allgemeiner Öffentlichkeit ausgehen, zerbrach diese Übereinstimmung im Verlauf der zweiten und dritten Welle; zunehmend breitete sich Misstrauen zwischen den verschiedenen gesellschaftlichen Kräften aus.

Zentral für dieses wachsende Misstrauen im Verlauf der Coronakrise ist die Art und Weise, wie über die Risiken der Pandemie und die notwendigen Maßnahmen kommuniziert wurde. Auftrag öffentlicher Institutionen zur Gesundheitsfürsorge und zur Gesundheitserhaltung ist es, Entscheidungen zu treffen und Maßnahmen zu erlassen, die das Leben, die Gesundheit und das Wohlergehen der Bürgerinnen und Bürger schützen und die Umwelt erhalten sollen. Die Entscheidungen müssen zum einen dem Kriterium der Effektivität im Sinne der nachweislichen Wirksamkeit entsprechen, zum anderen aber die Regeln und Kriterien der demokratischen Entscheidungsfindung im föderalen System der Bundesrepublik Deutschland einhalten [3]. Der Schutzauftrag muss so weit erfüllt werden, dass die zentralen gesellschaftlichen Werte, vor allem die, die im Grundgesetz festgelegt sind, nicht zur Disposition stehen [4, 5]. Beispiele dafür sind die Abwehr von Gesundheitsgefahren, die Einhaltung von Grenzwerten, die Vorsorge gegen katastrophale Ereignisse, aber auch nachvollziehbare Regeln für Abwägung zwischen gesundheitlichen, wirtschaftlichen und sozialen Zielsetzungen.

Gesellschaftliche Legitimität und Akzeptanz entstehen nicht durch einseitige Information oder durch öffentliche Belehrung. Legitimität und Akzeptanz entstehen durch verständigungsorientierte Dialoge, bei denen alle beteiligten Akteure gemeinsam lernen und nach tragbaren Handlungsoptionen suchen [6].

So viel zur Theorie. In der Praxis ist verständigungsorientierte Krisen- und Risikokommunikation keine leichte Aufgabe. In gesellschaftlichen Debatten über Risiken wie Infektionsschutz, Klimaschutz, Vorsorge gegen Unfälle oder Naturgefahren treffen die unterschiedlichsten Interessen und Anliegen zusammen. Oft erscheint es kaum möglich, einen gemeinsamen Nenner zu finden und sich über die Situation selbst und über mögliche Lösungswege zu verständigen. Meistens verschärfen sich die Konflikte im Verlauf der Debatte, man wirft sich gegenseitiges Unverständnis vor [7]. Viele öffentliche Institutionen geraten dabei zunehmend unter Druck.

Wie kommt man in dieser zugespitzten Situation zu einer vertrauensvollen Kooperation? Zentral für die Verständigung zwischen Institutionen der Risikovorsorge und des Risikomanagements und der Bevölkerung ist zum einen eine professionell und effektiv gestaltete Risikokommunikation, aber zum anderen die Schaffung und der Erhalt eines gegenseitigen Vertrauensverhältnisses zwischen den Akteuren und mit der Bevölkerung [8]. Im Folgenden werde ich mich vor allem auf die Schaffung und Wahrung einer Vertrauensbasis zwischen Institutionen des Risikomanagements und den von Risiken betroffenen Menschen und Gruppen konzentrieren. Die beiden wesentlichen Fragen, die ich im Sinne eines narrativen Reviews aus Literatur und eigener Kommunikationspraxis beantworten möchte, lauten:

Welche Faktoren sind maßgeblich dafür verantwortlich, ob und in welchem Ausmaß eine Vertrauensbasis und Glaubwürdigkeit geschaffen werden kann? Sowie: Wie lässt sich Vertrauen in Krisenzeiten schaffen bzw. erhalten?

## Vertrauen in öffentliche Institutionen

In Situationen mit hohem Unsicherheitsgrad und großem Konfliktpotenzial, in denen Entscheidungsprozesse schwierig und die Handlungsspielräume unübersichtlich werden, kann Kommunikation nur dann gelingen, wenn den Akteuren ein gewisses Grundvertrauen entgegengebracht wird [9]. Fehlt dieses Vertrauen, beherrschen Unterstellungen anstelle von offenem Austausch die Szene. Infolgedessen werden weder faktische Bezüge als Evidenz anerkannt, noch öffentliche Anweisungen oder Regeln befolgt. Entscheidungen der Behörden werden infrage gestellt und mit unlauteren Motiven wie Eigennutz oder Machtausdehnung in Verbindung gebracht [10]. Vertrauen oder Misstrauen bildet den Interpretationshintergrund jeglicher Krisen- und Risikokommunikation [11].

**Figure 1:**
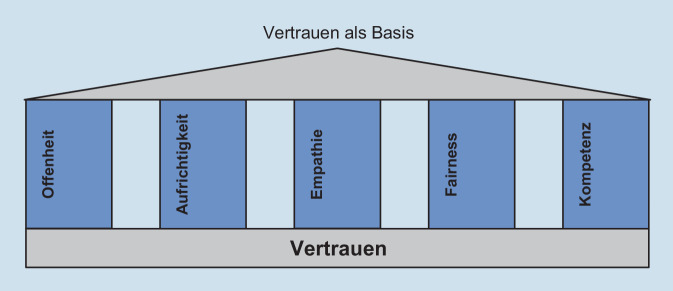

Vertrauen zu gewinnen und zu erhalten, ist ein permanenter Prozess wechselseitiger Erfahrungen. Es gibt aber durchaus Einflussgrößen, die gemeinsam eine vertrauensvolle Beziehung aufbauen oder stärken können [12, 13]. Eckpfeiler des Vertrauens sind Offenheit, Aufrichtigkeit, Empathie, Fairness und Kompetenz (Abb. 1 und Tab. 1). Diese Aspekte bestimmen weitgehend den Erfolg von Risikokommunikation. Sie beschreiben die interpersonalen Spielregeln, deren Verletzung Vertrauen gefährdet [14].

**Table 1:** 

*1. Offenheit:*
Bereitschaft, neue Themen und Sachverhalte aufzunehmen
Bereitschaft, sich mit allen denkbar betroffenen Akteuren auszutauschen
Bereitschaft, Entscheidungsprozesse und Handlungsoptionen transparent zu machen
Bereitschaft, sich mit anderen Weltbildern, Werten und Interessen auseinanderzusetzen
*2. Aufrichtigkeit*
Ehrlichkeit gegenüber allen am Entscheidungsprozess Beteiligten
Ehrlichkeit gegenüber der Öffentlichkeit
Ehrlichkeit im Nachweis von Informationsquellen und Informationsqualität
Ehrlichkeit in Bezug auf die eigene Gefühlslage
*3. Empathie*
Andere Menschen mit ihren Anliegen und Interessen prinzipiell ernst nehmen
Andere Perspektiven nachvollziehen und sich wechselseitig austauschen
Anderen Menschen Schutz und Unterstützung bei Gefahren gewähren
Anderen Menschen gegenüber Verantwortung übernehmen und sich für Fehler entschuldigen
*4. Fairness*
Klare Regeln der Informationsgewinnung, Zugänglichkeit der Informationen für alle betroffenen Anspruchsgruppen
Klare Regeln für Verfahren zur Chancen- und Risikokommunikation
Klare Regeln für den Entscheidungsprozess, nach welchen Prinzipien Handlungsoptionen ausgewählt werden oder Kompromisse entstehen
Klare Regeln für die kritische Prüfung und Bewertung von Prozess und Ergebnis
*5. Kompetenz*
Wissen über naturwissenschaftlich-technische Zusammenhänge von Risikofeldern
Wissen über Methoden zur Bewertung, zum Management und zur Kontrolle von Planungsvorhaben
Wissen über Konzepte und Verfahren zur Risikokommunikation
Wissen über gesellschaftliche Wertefragen, verschiedene Anspruchsgruppen mit ihren Wahrnehmungsmustern, Interessen und Präferenzen

Dazu kommt, dass institutionelles Vertrauen auch durch die dort agierenden Personen vermittelt wird. Vertrauen bedeutet, sich darauf verlassen zu können, dass die in Institutionen oder für beteiligte Institutionen agierenden Mitarbeiterinnen und Mitarbeiter persönlich verantwortlich handeln (zwischenmenschliches Vertrauen) und dass die Institutionen ihre erwarteten Leistungen erbringen (Systemvertrauen; [15]). Gerade wenn sich in Konflikten Vertreterinnen bzw. Vertreter von Organisationen miteinander verständigen können, um die aufgetretenen Konflikte gemeinsam zu bearbeiten, kann auch das Vertrauen in deren Organisationen wachsen [16].

Damit das gelingt, müssen die Sprecherinnen und Sprecher der jeweiligen Institutionen in ihren Handlungen und in ihren Äußerungen als *glaubwürdig* angesehen und bewertet werden.

## Glaubwürdigkeit als Grundlage von Vertrauen

Was versteht man unter Glaubwürdigkeit? Man bezeichnet eine Person oder eine Institution als glaubwürdig, wenn die Informationen, die man von dort erhält, nach bestem Wissen und Gewissen des Absenders wahr (den Tatsachen entsprechend) und wahrhaftig (die eigene Intention nicht verfälschend) sind. Eine glaubwürdige Quelle lügt nicht, verheimlicht nichts und bemüht sich, alle relevanten Aspekte und Standpunkte in die eigene Urteilsbildung und Kommunikation einzubeziehen [17]. Dies wurde schon in den Attributen in Tab. 1 angesprochen. In der Vergangenheit, vor allem in der Zeit vor der industriellen Revolution, waren die meisten Einsichten der Menschen durch persönliche praktische Erfahrungen geprägt [18]. Das meiste, was Menschen heute wissen, erfahren sie aus zweiter oder dritter Hand [19].^1^

Das bedeutet: Menschen sind in ihren Einsichten und Urteilen auf Informationen durch andere angewiesen. Sie können oftmals nicht aus eigener Erfahrung mögliche Bedrohungen einschätzen, die unter Umständen ihr Leben, ihre Gesundheit oder ihr Wohlergehen betreffen. Von daher muss man die Expertise von anderen, dafür spezialisierten Personen oder Institutionen einholen, die in der Lage sind, die betroffenen Personen vor diesen Gefahren zu warnen [20]. Bei vielen der heute vorherrschenden Risiken muss man sich aber auf eine große Bandbreite von Antworten gefasst machen. Zum Teil liegt das daran, dass in der stochastischen Analyse von Risiken eindeutige Antworten nicht zu erwarten sind (nur Bandbreiten mit Wahrscheinlichkeiten; [18, 21]). Zum Teil liegt es aber auch an den vermittelnden Botschaften, die vor allem durch die Medien transportiert werden. Journalistinnen und Journalisten neigen dazu, alle vorhandenen Urteile über eine Gefährdung nebeneinanderzustellen und ohne Prüfung auf deren wissenschaftliche Verlässlichkeit an die Medienkonsumentinnen und -konsumenten weiterzuleiten [22]. Somit stehen diese vor der Herausforderung, eine Fülle von möglichen Antworten auf die Fragen zu den Risiken unserer Zeit mental zu verkraften, die in der Regel von „völlig harmlos“ bis zu „höchst gefährlich“ reichen.

Da die meisten Menschen die Gültigkeit von Risikobewertungen nicht selber nachprüfen können, sind sie darauf angewiesen, die unterschiedlichen Quellen für die Risikoeinschätzungen nach ihrer Glaubwürdigkeit und Plausibilität zu beurteilen. Plausibilität ist allerdings kein guter Ratgeber, wenn der Sachverhalt komplex und die Beziehungsmuster zwischen Ursachen und Wirkungen unsicher sind [23]. Also verbleibt Glaubwürdigkeit als wichtigster Bezugspunkt.

Bei der Vergabe von Glaubwürdigkeit gibt es 3 Optionen: Man kann aus der Palette des breiten Angebots einer der Informationsquellen Glaubwürdigkeit beimessen und dann deren Interpretation des Sachverhaltes übernehmen. Hier liegt also eine Art Delegation an glaubwürdige Expertinnen und Experten vor. Die zweite Option ist generelles Misstrauen gegenüber allen. In diesem Fall ist eine Orientierung schwierig, weil man aus diesem prinzipiellen Entzug von Glaubwürdigkeit keine Handlungsmöglichkeiten ableiten kann. In dieser Situation neigen die meisten Personen dazu, das in der Botschaft angesprochene Risiko ganz zu vermeiden, oder sie wünschen sich, dass die Behörden die entsprechende Risikoquelle verbieten sollen [13]. Man kann dies als „Sehnsucht nach Nullrisiko“ bezeichnen.

Die dritte Option ist, nach Anzeichen dafür zu suchen, welcher Informationsquelle man das meiste Vertrauen schenken kann. Da man den Inhalt der jeweiligen Aussagen nicht beurteilen kann, lässt sich die Glaubwürdigkeit nur an sogenannten peripheren Merkmalen ablesen.^2^ Periphere Merkmale sind Aspekte, die mit der Informationsquelle assoziativ verbunden werden und die einem indirekt darüber Aufschluss geben sollen, ob die Quelle als glaubwürdig einzustufen ist oder nicht [26]. Solche Merkmale können sein: vermutete Interessengebundenheit, rhetorische Fähigkeiten, sympathisches Auftreten, Anknüpfung an vertraute Lebensverhältnisse oder Wertvorstellungen, aber auch so triviale Dinge wie Kleidung, Gestik und Aussehen.

Viele dieser peripheren Merkmale sind professionellen Kommunikationsberaterinnen und -beratern durchaus bekannt. Sie trainieren ihre Klientinnen und Klienten darauf, möglichst viele Merkmale zugeschriebener Glaubwürdigkeit auszustrahlen. Das Problem ist aber, dass die Zuschreibung von Glaubwürdigkeit aufgrund peripherer Merkmale nur selten mit dem Wahrheitsgehalt der damit verbundenen Aussagen übereinstimmt. Periphere Merkmale sagen nämlich wenig oder oft auch gar nichts über die Gültigkeit und Nachprüfbarkeit der jeweils vorgenommenen Argumente aus.

Es gibt also 3 Optionen, auf Informationen über Botschaften zu reagieren, für deren Beurteilung Menschen keine eigenen Erfahrungswerte besitzen: a) Sie halten keine Quelle für glaubwürdig, b) sie halten die Quelle für glaubwürdig, der sie in der Vergangenheit besonderes Vertrauen entgegengebracht haben, und c) sie sprechen Glaubwürdigkeit mal der einen und mal der anderen Quelle zu, je nach Vorliegen der peripheren Merkmale [27].

Alle 3 Reaktionsweisen sind in unserer Gesellschaft immer wieder anzutreffen [13]. Zum einen gibt es Menschen, die aufgrund tiefgreifender Beziehungen ein unerschütterliches Vertrauen in bestimmte Gruppen und Institutionen haben. Sie sind vielleicht seit Langem Mitglied einer Gewerkschaft und nehmen das als glaubwürdig auf, was als offizielle Verlautbarung der Gewerkschaft kommuniziert wird. Dadurch erleben sie selten kognitive Dissonanzen, weil sie nur ihren eigenen Bezugsgruppen und sonst niemandem vertrauen [28]. Der prozentuale Anteil der Personen mit einer hohen Loyalität zu einer Bezugsgruppe geht jedoch seit Jahren stetig zurück, das kann man zum Beispiel an der zunehmenden Zahl von Wechselwählerinnen und -wählern ablesen.^3^ Möglicherweise steigt aber an den Rändern der Gesellschaft die Loyalität zu bestimmten – meist ideologisch oder weltanschaulich festgelegten – Positionen wieder an. Populistinnen und Populisten greifen bewusst auf die Verunsicherung durch fehlende Loyalitäten zu den herkömmlichen Institutionen wie Wissenschaft, Politik oder Behörden zurück und versprechen Geborgenheit und Sicherheit, wenn man sich ihnen nur anvertraut [29].

Dieser Gruppe entgegengesetzt ist ein Personenkreis, der niemandem mehr vertraut. Dieser gewinnt mehr und mehr an Zulauf. Er versammelt verunsicherte bis zynisch eingestellte Menschen, die das Grundvertrauen in Gruppen und Institutionen der Wissensvermittlung verloren haben und nur noch Spott und Verachtung für die wirtschaftlichen und politischen Institutionen übrighaben [30]. Damit geht ein hohes Maß an Politikverdrossenheit einher [31]. Interessanterweise ist es gerade dieser Personenkreis, der gegenüber radikalen politischen Parolen und populistischen Versprechungen häufig anfällig wird, mit der Konsequenz, dass die beteiligten Personen dann doch Vertrauen gegenüber einer Gruppe wie etwa radikalen Populistinnen und Populisten aufbauen. Oft enthalten Angebote aus dem Kreis der populistischen Meinungsmacherinnen und -macher einige wahre, aber von der Politik oder anderen Gruppen verschwiegene Aussagen, die dann als Köder dienen, um ein ganzes Narrativ von Interpretationen bis hin zu Verschwörungstheorien zu rechtfertigen.

Die überwiegende Anzahl der Menschen verfolgt aber die dritte Option und verteilt Glaubwürdigkeit je nach Situation, Kontext und der selektiven Aufnahme von peripheren Merkmalen [27]. Genau diese Flexibilität in der Zuschreibung von Glaubwürdigkeit führt zur persönlichen und dann auch kollektiven Verunsicherung. Die betroffenen Personen erleben fortwährend Situationen von kognitiver Dissonanz, weil die gerade wirksamen peripheren Merkmale nicht immer den Informationsinhalten Glaubwürdigkeit verleihen, welche die eigene (vorgefasste) Haltung oder Meinung bestätigen. Und dieses Karussell kann sich dann immer wieder in beide Richtungen drehen. Die Folge einer solchen vagabundierenden Zuteilung von Glaubwürdigkeit an die jeweils eine oder andere Partei ist wachsende Verunsicherung über das, was wirklich stimmt. Wenn es sich dabei um Bedrohungen handelt, die auch für einen selbst relevant sein können, erwächst aus der Verunsicherung Angst [32]. Gerade dann ist aber eine vertrauensbildende Kommunikation unerlässlich, denn ein diffuses Gefühl von Angst verhilft nicht zu neuen Einsichten oder Erfahrungen, sondern lähmt einen Menschen in seinen Überlegungen und Handlungen.

## Risikokommunikation: Herausforderungen und Lösungsansätze

Für ein erfolgreiches Risiko- und Krisenmanagement sind also Vertrauen und Glaubwürdigkeit essenziell. In Zeiten der Krise, wie etwa während der Coronapandemie, ist eine effektive Bewältigung der Bedrohungssituation ohne eine vertrauensvolle Beziehung zwischen Behörden und Bevölkerung kaum möglich. Das Vertrauen der Öffentlichkeit in die Problemlösungskompetenz und Gemeinwohlausrichtung der Behörden ist auschlaggebend, um zum einen Ängste und Fehleinschätzungen der Risiken zu vermeiden und zum anderen Zustimmung zu den erforderlichen Maßnahmen (Compliance) zu gewinnen [33]. Letzter Punkt betrifft auch die Zusammenarbeit mit den Medien, die den Informationen der öffentlichen Institutionen vertrauen sollten. Wird in den Medien Misstrauen gegenüber den verantwortlichen Risikomanagern geschürt, so verschärft sich die Krise [34]. Vertrauen muss also bereits vorhanden sein, bevor eine Krise eintritt und öffentlich wirksam wird.

Vertrauen aufzubauen und zu erhalten ist im Rahmen komplexer Krisen wie der einer Pandemie nicht einfach zu bewerkstelligen. Die Chancen von Impfungen und die Risiken, die damit verbunden sind, abzuschätzen ist erstens hochkomplex hinsichtlich der zugrunde liegenden wissenschaftlichen Zusammenhänge. Zweitens ist die Datenbasis, die zur Erstellung von kausalanalytischen Ursache-Wirkungs-Ketten dient, unsicher oder es besteht Ungewissheit über Interdependenzeffekte [35]. Drittens berühren solche Krisen nicht nur technische oder ökonomische Problemfelder, sondern werfen auch moralisch-ethische Fragestellungen auf [36].

Gelingende Kommunikation ist dabei von 3 Rahmenbedingungen abhängig: Zum ersten müssen die Botschaften die Zielgruppe auch erreichen und von ihr verstanden werden, zum zweiten müssen die Empfängerinnen und Empfänger bei aller Pluralität von Meinungen und Urteilen aus den Botschaften deutlich ableiten können, welche Handlungsmöglichkeiten zur Verfügung stehen. Zum dritten müssen sie diese Handlungsgebote auch als sinnvoll und angemessen einstufen, damit sie auch in die Realität umgesetzt werden. Diese 3 Bedingungen sind nicht leicht zu erfüllen [37]. Eine weitere Erhöhung der Papierflut oder die Vervielfachung von öffentlichen Veranstaltungen wird dieses Problem nicht lösen helfen. Es gibt 2 Strategien, die einen möglichen Ausweg aus dieser schwierigen Situation zeigen: Einbindung sozialer Netzwerke und Einbindung der Zielgruppen in partizipative Verfahren [9].

### Einbindung sozialer Netzwerke.

Unabhängig davon, um welche Krise es sich handelt, gibt es immer Organisationen oder Gruppen, um die sich soziale Netzwerke ausgebildet haben. Solche Netzwerke können religiöse Gemeinden, Vereine, Nachbarschaftsgruppen oder Freizeitgruppen sein. Wenn es gelingt, diese Netzwerke aktiv in die Kommunikationsarbeit und in Dialoge über Risiken einzubinden, kann man relativ sicher sein, dass zumindest innerhalb des Netzwerkes ein intensiver Kommunikationsaustausch stattfindet, der gleichzeitig Vertrauen aufbauen kann. Denn wenn sich die jeweils kommunizierenden Netzwerkpartnerinnen und -partner mit ihren Mitgliedern austauschen, stehen sie offenkundig hinter den Botschaften und bilden eine Allianz mit dem ursprünglichen Kommunikator bzw. der ursprünglichen Kommunikatorin. Empirische Untersuchungen weisen darauf hin, dass Institutionen, die beginnen, mit anderen Organisationen oder Gruppen mit hohem Vertrauenspotenzial zusammenzuarbeiten, davon mitprofitieren, indem sie unter anderem als glaubwürdiger eingestuft werden als vor der Kooperation [38].

### Einbindung der Zielgruppen in partizipative Verfahren.

Lernprozesse werden selten dadurch ausgelöst, dass Behörden Informationen an Personen in Form von Broschüren oder Leitfäden weiterleiten. Solche Informationsangebote können Kommunikation unterstützen und als Erinnerung für Handlungsanleitungen dienen, aber sie sind kaum geeignet, kritisch eingestellte Menschen von etwas zu überzeugen, an das sie nicht glauben oder das sie in Zweifel ziehen [39]. Selbst interaktive Formate mit Rückfragen von Bürgerinnen und Bürgern an Behörden oder Gremien reichen kaum aus, um bei den Teilnehmenden ein Gefühl von Glaubwürdigkeit und Vertrauenswürdigkeit auszulösen. Häufig nehmen die Diskutantinnen und Diskutanten nur Argumente auf, die die eigene Einstellung unterstützen [40]. Verändert man aber die Rahmenbedingungen und gibt den Teilnehmenden an solchen Foren die Gelegenheit der eigenen Gestaltung (etwa in Form der Mitwirkung an Katastrophenplänen, Maßnahmen zur Krisenbewältigung oder Erarbeitung von Handlungsempfehlungen), werden die betroffenen Bürgerinnen und Bürger selbst zu Planerinnen und Planern und identifizieren sich mit den Aufgaben der Behörden [41]. Partizipation schafft aktive Lernräume und Gelegenheiten zur emotionalen Identifikation mit den Aufgaben des Krisenmanagements. Damit dies wirksam wird, muss aber sichergestellt sein, dass auch wirklich Gestaltungsspielräume existieren. Nur Ja-Nein-Entscheidungen führen eher zur Polarisierung als zur Befriedung in konfliktreichen Entscheidungssituationen [42].

Trotz aller kommunikativen und partizipativen Maßnahmen wird es wohl niemals gelingen, alle potenziell Betroffenen einer Krise sachgemäß zu informieren oder sogar diese in die Planungen aktiv einzubinden. Aber je mehr Individuen an den Planungen und Vorbereitungen beteiligt sind, desto eher ist mit einer breiten Zustimmung und vor allem mit einer breiten Mitwirkung im Ernstfall zu rechnen.

## Diskussion: Vertrauensaufbau und -verstetigung

Zu Beginn dieses Artikels standen 2 Fragen: Welche Faktoren sind maßgeblich dafür verantwortlich, ob und in welchem Ausmaß eine Vertrauensbasis und Glaubwürdigkeit geschaffen werden können? Sowie: Wie lässt sich Vertrauen in Krisenzeiten schaffen bzw. aufrechterhalten?

Bei der ersten Frage zeigen die Ausführungen in diesem Beitrag, dass vor allem die Faktoren Offenheit, Aufrichtigkeit, Empathie, Fairness und Kompetenz den Erfolg von Risikokommunikation weitgehend bestimmen. Ob allerdings ein Kommunikationsangebot als offen, fair, empathisch und kompetent wahrgenommen wird, hängt nicht nur von den Inhalten und der Form der Kommunikation ab, sondern auch von der Glaubwürdigkeit der Institution selbst. Diese ist in einem sozialen Klima von Vertrauen oder Misstrauen gegenüber Institutionen allgemein und durch historische Kontextbedingungen gewachsen und lässt sich häufig durch Kommunikation nur marginal beeinflussen. Hier sollten neben kommunikativen Verfahren auch Prozesse der aktiven Mitwirkung der Stakeholder und der betroffenen Bürgerschaft an der Gestaltung und Umsetzung staatlicher Maßnahmen zum Gesundheitsschutz organisiert werden. Zur Bewältigung der Coronakrise hat zum Beispiel Baden-Württemberg einen eigenen Bürgerrat ins Leben gerufen, in dem zufällig ausgewählte Bürgerinnen und Bürger die Landesregierung bei der Konzipierung und Umsetzung der Schutzmaßnahmen beraten^4^. Diese Rückkopplung mit der Bürgerschaft ist ein wichtiges Instrument, um die notwendige Glaubwürdigkeit der Landesregierung zu unterstützen.

Bei der zweiten Frage nach den Erfolgskriterien für eine gelingende Risikokommunikation können die genannten Merkmale wie Offenheit, Aufrichtigkeit, Empathie, Fairness und Kompetenz als Leitorientierungen für die Gestaltung von Kommunikationsangeboten dienen. Wesentlich ist aber, dass eine Kongruenz zwischen den Erwartungen der betroffenen Menschen und den erlebten Leistungen der entsprechenden Institutionen besteht. Allerdings sind die Leistungen von Entscheidungsträgerinnen und -trägern fortwährend dem Dauerfeuer der öffentlichen Kritik ausgesetzt. Denn Komplexität und Mehrdeutigkeit der Datenlage sowie Pluralität der Werte und Präferenzen lassen eine eindeutige Bewertung und Abwägung nicht zu [43]. Um trotz dieser Barrieren eine glaubwürdige Informationspolitik zu gestalten, erscheinen mir folgende Maßnahmen und Vorkehrungen für Institutionen der Gesundheitsvorsorge und des Gesundheitsschutzes besonders empfehlenswert:

### Wissenschaftliche Evidenz als Maßstab nehmen.

Es gilt, von der postmodernen Vorstellung Abschied zu nehmen, dass Wissen in beliebiger Form sozial konstruiert sei und es keine übergreifenden Qualitätsansprüche oder Kriterien für Wahrheitsansprüche gäbe. Die jüngst entflammte Diskussion um „Fake News“ (manipulativ verbreitete, vorgetäuschte Nachrichten), um Echokammern und um postfaktische Kommunikation ist ein beredtes Zeugnis dafür, dass bei einem Vakuum an wissenschaftlich gesichertem Wissen die Lücke mit Halbwissen und interessengeleiteten Überzeugungen gefüllt wird.^5^ Die Realität ist aber: Menschen sterben und leiden aufgrund von falschem Wissen. Die Coronapandemie hat dies allen vor Augen geführt: Diejenigen, die lauthals behauptet haben, dass das Virus SARS-CoV‑2 nicht existieren würde oder harmlos wäre, haben das Leben von vielen Menschen aufs Spiel gesetzt. Gerade weil in komplexen Situationen zuverlässiges Wissen geradezu „lebenswichtig“ ist, ist es dringend erforderlich, so eindeutig wie möglich die Grenzen der Bandbreite legitimen Wissens zu bestimmen. Hier müssen Wissenschaftsorganisationen, risikoregulierende Behörden und Medien gemeinsam dafür einstehen, dass nur auf Evidenz aufgebaute Behauptungen über Risiken als legitim und als handlungsorientierend anerkannt sein dürfen. Handlungsleitende Aussagen dürfen nicht den anerkannten Erkenntnissen über faktische Zusammenhänge widersprechen.

### Expertenwissen und Laienbeurteilungen integrieren.

Die Gefahrenabschätzung von Fachleuten und die Risikowahrnehmung von Laien sind eher als gegenseitige Ergänzung denn als Konkurrenz zu sehen. In den Coronadebatten der beiden letzten Jahre hat sich gezeigt, dass die Vertreterinnen und Vertreter der Öffentlichkeit den Stimmen aus der Wissenschaft wie der jeweiligen Fachbehörden (z. B. Robert Koch-Institut) überwiegend hohe Glaubwürdigkeit zugesprochen haben [44]. Aber auch bei voller Einsicht in wissenschaftlich belegte Zusammenhänge verbleibt die politische Wertentscheidung, nach welchen Kriterien und nach welchem Maßstab die zu erwartenden positiven und negativen Folgen von Maßnahmen oder Regelungen zu beurteilen sind und wie eine angemessenen Güterabwägung der zur Wahl stehenden Maßnahmen vorgenommen werden soll. Das setzt vor allem hohe Transparenz über die Kriterien der Abwägung („trade-off“) und Verständnis für andere wertbezogene Priorisierung voraus.

### Kommunikationsangebote optimieren.

Neben diesen grundlegenden Empfehlungen können auch einige instrumentelle Empfehlungen gegeben werden [45]:

- Zugänge zu wichtigen Informationen dort verfügbar machen, wo sich die jeweiligen Zielgruppen real oder virtuell aufhalten (etwa am Arbeitsplatz, an Sportstätten, an Freizeiteinrichtungen, in bestimmten Internetforen und Portalen);
- Argumente, Illustrationen und Gedankenführung den aus Risikowahrnehmungsstudien bekannten Mustern anpassen und adressatengerecht aufarbeiten;
- wenn möglich, Handlungsalternativen anbieten, aus denen man auswählen kann (erhöht u. a. das eigene Selbstwertgefühl);
- Mitstreiterinnen und Mitstreiter suchen und diese überzeugen, als öffentlich wirksame Botschafterinnen und Botschafter der wesentlichen Aussagen aufzutreten. Dabei kommt es darauf an, Personen zu wählen, die gerade bei den jeweiligen Zielgruppen hohe Glaubwürdigkeit genießen: Personen aus unterschiedlichen Kulturkreisen, Repräsentantinnen und Repräsentanten alternativer Medizin und naturnaher Heilmethoden oder Angehörige von zivilgesellschaftlichen Gruppierungen.

## Fazit

Gelingende Risikokommunikation ist also an Konzepte, Verfahren und Praktiken gebunden, die sicherstellen, dass die Botschaften überzeugende wissenschaftliche Evidenz vermitteln, Offenheit für Anliegen der Zielgruppen signalisieren sowie durch eine gelungene Synthese von Wissen und Handlungsorientierung Glaubwürdigkeit und Kompetenz ausstrahlen. Angesichts der Unsicherheit von Wissen und der Unübersichtlichkeit der Kommunikationsvorgänge in der Gesellschaft ist diese Aufgabe nicht leicht zu erfüllen. Dazu kommt die Erschwernis einer virtuellen Kommunikationslandschaft, die eher auf Gesinnungsbestätigung als auf sachliche Information setzt. Trotz dieser Schwierigkeiten können Institutionen der Risikobewertung und -regulierung durch geschickte Koalitionsbildung mit Organisationen und Gruppen mit hohem Vertrauenspotenzial, durch transparente, ergebnisoffene Formen der Kommunikation und durch die Einbindung von Stakeholdern und betroffenen Individuen in das Risikomanagement Vertrauen aufbauen und über die Zeit verstetigen.

## Footnotes

1 „Den Insassen der Wissensgesellschaft falle es deutlich schwerer als früher, Erfahrungen zu sammeln: Der Einzelne hat zwar jede Menge Erlebnisse, aber es gelingt ihm nicht mehr, diese Erlebnisse in Erfahrungen zu verwandeln, in eine kohärente ‚Erzählung‘ des eigenen Lebens. Doch ohne einen festen Erfahrungskern reagieren die Menschen nur noch reflexhaft und situativ – und eben nicht mehr ‚klug aus Erfahrung‘. Was dann an Lebenserfahrung noch übrig bleibt, das droht durch technische Entwicklungen und Innovationen rasend schnell entwertet zu werden. Das Subjekt der Erfahrung sieht plötzlich ‚sehr alt‘ aus“. Aus: Assheuer, T. (2012): Worauf ist noch Verlass? *ZEIT Online*, 03.05.2012. http://www.zeit.de/2012/19/PS-Erfahrung-Philosophie (abgerufen am 04.11.2021).

2 Der Ansatz der peripheren Urteilsbildung geht auf das sogenannte Elaboration-Likelihood-Modell von Petty und Cacioppo [24] zurück, die Urteilsbildungsprozesse nach 2 Routen unterscheiden: den zentralen Ansatz und den peripheren Ansatz. Speziell zu den peripheren Merkmalen siehe: [25].

3 „In den westlichen Bundesländern ist eher eine allmähliche Abschwächung und teilweise sogar eine Auflösung längerfristiger Parteibindungen zu beobachten. Zum einen hat sich bei einem Teil der Wählerinnen und Wähler zunehmend eine distanziertere Haltung gegenüber den Parteien herausgebildet. Zum anderen hat sich aber auch die Angebotsstruktur geändert. Das Aufkommen neuer Parteien und Koalitionsoptionen hat fast zwangsläufig zu einer Umorientierung auf dem Wählermarkt geführt. Traditionelle Loyalitäten, wie sie sich besonders in der Frühphase der Bundesrepublik Deutschland ausgebildet hatten, verlieren damit tendenziell ihren bislang beherrschenden Einfluss auf die Wahlentscheidung.“ Aus: Korte, C.-R. (2009): *Parteienbindung und Kandidatenorientierung.* Bundeszentrale für politische Aufklärung, http://www.bpb.de/politik/wahlen/bundestagswahlen/62629/parteibindung-und-kandidaten (abgerufen am 04.11.2021).

4 https://www.baden-wuerttemberg.de/de/service/presse/pressemitteilung/pid/buergerforum-corona-erfolgreich-gestartet/ (abgerufen am 29.12.2021).

5 „Der sogenannte Echo-Chamber-Effekt verstärkt Nachrichten vor allem innerhalb jener Gruppen, die ohnehin einer bestimmten Ideologie oder politischen Richtung anhängen. Ob TTIP-Gegner oder Finanzmarktkritiker, ob Pegida-Anhänger oder Neokonservative: Sie alle abonnieren auf Facebook oder anderen sozialen Medien vor allem jene Kanäle, deren Feeds ihre Sicht der Dinge bestätigen. Warum dieses System so gut funktioniert? Weil der Mensch nichts so sehr hasst, wie kognitive Schieflagen, den Zweifel, das Infragestellen der eigenen Position. Er fühlt sich am wohlsten, wenn er sich – eben wie in einem Echoraum – mit seiner Meinung in guter Gesellschaft, in einer gefühlten Mehrheit glaubt.“ Aus: Ziener, M. (2016) Wohlfühlen im Echoraum. *Newsletter der IPG* (International Politik und Gesellschaft) vom 09.05.2016, o.S. http://www.ipg-journal.de/kommentar/artikel/wohlfuehlen-im-echoraum-1416.
